# Serotonergic Dysfunction and the Interacting Role of TAR DNA-Binding Protein 43 in the Progression of Amyotrophic Lateral Sclerosis: A Scoping Review

**DOI:** 10.3390/biom16101470

**Published:** 2026-10-09

**Authors:** Lauren L. Hutchison, Makenna M. Parkes, Erik P. Pioro

**Affiliations:** 1 Neuroscience Graduate Program, Faculty of Medicine, University of British Columbia, 2215 Wesbrook Mall, Rm 3402, Vancouver, BC V6T 1Z3, Canada; lauren.hutchison@ubc.ca; 2 MD Undergraduate Program, Faculty of Medicine, University of British Columbia, 2194 Health Sciences Mall, Rm 317, Vancouver, BC V6T 1Z3, Canada; mparkes2@student.ubc.ca; 3 Djavad Mowafaghian Centre for Brain Health, Department of Medicine (Neurology), University of British Columbia, 2215 Wesbrook Mall, Rm 3439, Vancouver, BC V6T 1Z3, Canada

**Keywords:** amyotrophic lateral sclerosis (ALS), serotonin (5-HT), serotonin receptor (5-HTR), serotonergic dysfunction, TAR DNA-binding protein 43 (TDP-43), protein aggregation

## Abstract

Amyotrophic lateral sclerosis (ALS) is a progressive neurodegenerative disease characterized by the loss of motor neurons and widespread neural dysfunction. Loss of serotonin (5-HT) signaling and TAR DNA-binding protein 43 (TDP-43) dysfunction have both been identified as contributing factors to disease progression, although their relationship has yet to be fully defined. The objective of this review was to assess whether any mechanistic links exist between serotonergic dysfunction and TDP-43 proteinopathy in ALS. A scoping review was conducted in accordance with Preferred Reporting Items for Systematic Reviews and Meta-Analyses extension for Scoping Reviews (PRISMA-ScR) guidelines. Databases were queried for the peer-reviewed literature concerning ALS pathophysiology, 5-HT, and TDP-43 (1946 to January 2026). Articles were screened and data independently extracted by two reviewers, which were subsequently synthesized qualitatively (*n* = 14). The current literature suggests that serotonergic dysfunction can be identified early in ALS pathology, indicated by decreased 5-HT concentration, degeneration of raphe projections, and changes in receptor activity. TDP-43 proteinopathy is a defining characteristic of ALS progression, characterized by cytoplasmic mislocalization and aggregation of TDP-43. Additionally, studies have identified bidirectional links between these dysfunctions: TDP-43 impairs serotonergic transmission, and 5-HT deficiency may drive pathological TDP-43 aggregation. Many mechanisms affected by both serotonergic and TDP-43 dysfunction are associated with disease progression including excitotoxicity, metabolic disturbances, and network hyperexcitability. Despite this, there is a lack of direct experimental evidence elucidating connections between these pathologies. Serotonergic dysfunction and TDP-43 proteinopathy may be linked by shared mechanisms in ALS, though specific causality has not been identified. Further research should aim to clarify the connection between these processes and discover therapeutic targets.

## 1. Introduction

### 1.1. Rationale

Amyotrophic lateral sclerosis (ALS), also known as Lou Gehrig’s disease, is a progressive neurodegenerative disorder characterized by the degeneration of upper and lower motor neurons, leading to muscle weakness, atrophy, paralysis, and ultimately respiratory failure [1]. Disease onset is heterogeneous, although it is most commonly classified as limb- or bulbar-onset, and only rarely as respiratory-onset ALS. Although traditionally considered a motor disorder, up to 50% of patients exhibit cognitive and behavioural impairments, including executive dysfunction, aphasia, and mood disturbances, indicating the involvement of non-motor brain regions [2,3]. ALS pathogenesis remains poorly understood. Approximately 10% to 15% of cases are familial (fALS), associated with mutations in genes such as *C9orf72*, *SOD1*, *TARDBP*, and *FUS* [4], while the majority are sporadic (sALS), with no single clearly defined monogenic cause. Environmental factors, including toxin exposure, military service, smoking, and certain occupational exposures, have also been implicated in ALS risk, although their mechanisms remain unclear. Neurodegeneration in ALS may extend beyond the motor cortex to regions other than frontotemporal lobes, such as the basal ganglia, overlapping with patterns observed in Huntington’s and Parkinson’s diseases [5].

A central and increasingly well-defined feature of ALS pathology is protein aggregation, particularly involving the 43 kDa TAR DNA-binding protein (TDP-43). TDP-43 proteinopathy is observed in both fALS and sALS and is thought to “bridge the divide” between these forms of the disease. The few notable exceptions include *SOD1*- and *FUS*-linked ALS, in which TDP-43 proteinopathy appears to be absent [6]. Similar to other neurodegenerative disorders, ALS may involve prion-like spread of misfolded proteins [7]. TDP-43 is tightly regulated, with both depletion and overexpression inducing toxicity [8]. Critically, its mislocalization from the nucleus to the cytoplasm is strongly associated with neuronal toxicity and cell death, with cytoplasmic abnormal TDP-43 serving as a key predictor of toxicity [9]. Despite this, the mechanisms driving TDP-43 mislocalization, aggregation, and propagation remain unclear.

Excitotoxicity is another major contributor to ALS pathology. Glutamate, the primary excitatory neurotransmitter in the central nervous system (CNS), activates N-methyl-D-aspartate (NMDA) and α-amino-3-hydroxy-5-methyl-4-isoxazolepropionic acid (AMPA) receptors to regulate neuronal firing. However, excessive or dysregulated glutamatergic signaling can lead to toxic intracellular calcium accumulation and cell death. This occurs through either elevated extracellular glutamate (“classical” excitotoxicity) or increased neuronal sensitivity to normal glutamate levels (“slow” excitotoxicity) [10]. Given the degeneration of both cortical and spinal motor neurons, excitotoxicity is thought to play a broad role in ALS. Therapeutic support for this hypothesis comes from the success of riluzole, which reduces glutamate toxicity via enhanced glutamate clearance from synaptic clefts [11]. Additionally, dextromethorphan/quinidine (DM/Q), marketed in the United States as Nuedexta^®^, is a treatment for pseudobulbar affect (PBA), which occurs not infrequently in ALS. The active ingredient, DM has been found to act as a noncompetitive NMDA receptor antagonist, thereby reducing glutamatergic firing and potentially dampening glutamate-mediated excitotoxicity [12]. However, other treatments, such as edaravone (Radicava^®^), act independently of glutamate (potentially as free radical scavengers), suggesting that excitotoxicity is only one component of a multifactorial disease process [13].

In addition to glutamatergic dysfunction, serotonergic signaling has emerged as a potential contributor to ALS pathogenesis. Serotonin (5-HT) interacts with multiple neurotransmitter systems, including GABAergic, glutamatergic, and dopaminergic pathways [14,15]. Serotonergic projections in the CNS originate primarily from the raphe nuclei (RN) in the brainstem and extend widely to regions involved in motor control, cognition, and emotional regulation, including the cortex, striatum, substantia nigra, and ventral tegmental area [16]. Dysfunction in these pathways may contribute to both motor and cognitive symptoms in ALS and further exacerbate excitotoxic damage by disrupting the balance between excitatory and inhibitory signaling [17]. Notably, extensive pathology in lower RN has been reported in ALS, while the upper RN appears to be only minimally affected by TDP-43 pathology [17]. Taken together, these observations raise the possibility of a modulatory relationship between the serotonergic system and TDP-43 pathology in ALS.

### 1.2. Objectives

This potential relationship between serotonergic dysfunction and TDP-43 proteinopathy in ALS remains poorly understood. While each of these factors has been independently implicated in disease onset and progression, it is unclear whether, or to what extent, they interact as part of a unified pathogenic mechanism. The relationship between serotonergic signaling and TDP-43 mislocalization and aggregation has not yet been systematically examined.

The primary objective of this study was to conduct a scoping review of the existing literature to evaluate potential mechanistic links between 5-HT and TDP-43 abnormalities in ALS. This included assessing whether serotonergic dysfunction may influence TDP-43 pathology directly or indirectly through modulation of excitotoxic pathways, neuronal hyperexcitability, neuroinflammation, and broader neurotransmitter imbalances.

A secondary objective was to identify common molecular or cellular pathways that may link these processes, with particular attention to calcium signaling, neuronal excitability, and protein homeostasis. By synthesizing findings across genetic, molecular, and systems-level studies, this review aimed to clarify whether these mechanisms converge on common pathogenic processes.

This work also sought to identify gaps in the current literature and highlight promising directions for future experimental research. A clearer understanding of the interaction between serotonergic signaling and TDP-43 pathology may also contribute to the identification of novel therapeutic targets for ALS, particularly those aimed at modulating neurotransmitter systems or preventing protein mislocalization and aggregation.

## 2. Methods

In this paper, we performed a scoping review to examine the existing literature on the relationship between 5-HT, TDP-43, and the etiology and pathogenesis of ALS. While no formal protocol was prospectively registered, our scoping review methodology was developed in accordance with the Preferred Reporting Items for Systematic Reviews and Meta-Analyses extension for Scoping Reviews (PRISMA-ScR) checklist [18] (see Supplementary File S1).

### 2.1. Eligibility Criteria

The authors developed predefined inclusion and exclusion criteria, which were reviewed by the corresponding author. To be included in this review, documents were required to include a substantial explanation of the heritability, etiology and pathophysiological mechanisms underlying ALS. Additionally, documents were required to include, at minimum, a section discussing 5-HT, the serotonergic system, and/or any 5-HT precursors and their interaction with ALS. Finally, documents were required to mention of TDP-43’s role in ALS and/or how TDP-43 interacts with 5-HT or the serotonergic system in ALS.

Papers were included in the scoping review analysis if they were peer-reviewed, published between 1946 and 2026, written in English, and met all three criteria developed by the authors. Papers were excluded if they did not meet all three predefined criteria, or if one or more criteria were mentioned without substantive discussion or sufficient detail to permit meaningful interpretation. Articles that provided context but did not meet the eligibility criteria were utilized strictly as background literature and were excluded from data charting.

### 2.2. Information Sources

To identify potentially relevant documents, search strategies were developed in collaboration with an experienced Dentistry-Medicine reference librarian and refined over several meetings. The final search strategy utilized controlled vocabulary (MeSH and Emtree) terms related to ALS, 5-HT, and TDP-43. The full search strategy is provided in Supplementary File S2.

Using the finalized search strategy, the following databases were searched from 1946 to 28 January 2026: Ovid MEDLINE(R) ALL, Embase, EBM Reviews—Cochrane Database of Systematic Reviews, EBM Reviews—Cochrane Central Register of Controlled Trials, Health and Psychosocial Instruments, EBM Reviews—Cochrane Methodology Register, EBM Reviews—Database of Abstracts of Reviews of Effects, EBM Reviews—Health Technology Assessment, EBM Reviews—NHS Economic Evaluation Database, EBM Reviews—ACP Journal Club, EBM Reviews—Cochrane Clinical Answers, BIOSIS Previews, and JBI Best Practice. These databases primarily captured the peer-reviewed literature, with limited inclusion of the grey literature (e.g., conference abstracts and reports, occasional preprints), but a systematic search of the grey literature sources was not conducted. This OVID search was supplemented by manual review of seminal authors’ bibliographies and by screening relevant papers. Final search results were imported into Covidence systematic review software (Veritas Health Innovation, Melbourne, Australia, available at www.covidence.org; accessed on 28 January 2026) for screening. Titles and abstracts were independently screened by two reviewers, followed by full-text assessment of potentially eligible studies. The study selection and screening process were conducted in accordance with PRISMA-ScR guidelines. Screening was performed independently by two reviewers, with discrepancies resolved through discussion.

### 2.3. Data Charting

Data charting was performed by two reviewers working independently and blinded to each other’s input using Covidence systematic review software. A custom template was used to extract information from the 14 included studies. Following individual data charting, the two reviewers compared their extracted data, and any discrepancies were resolved through discussion until consensus was reached. Extracted data included general study information (e.g., title, study ID, lead author contact details, and date of publication), study characteristics (e.g., aim of study/research question, study design, participant information), as well as results and outcomes related to each of our criteria (e.g., 5-HT relationship with ALS, TDP-43 relationship with ALS, 5-HT relationship with TDP-43).

### 2.4. Synthesis

The included studies were grouped according to the primary thematic relationship emphasized within each paper (e.g., 5-HT and ALS, TDP-43 and ALS, or 5-HT and TDP-43). Within each group, we summarized study characteristics and broad findings and identified patterns and differences. The evidence was synthesized qualitatively and presented in narrative form.

## 3. Results

Across the included studies, evidence suggested potential relationships among 5-HT, TDP-43 prion-like propagation, and ALS clinical features. These findings support the need for further research into the connections among 5-HT, TDP-43 propagation, and ALS phenotypes, which may help identify future therapeutic targets.

### 3.1. Selection of Sources of Evidence

**Figure 1:**
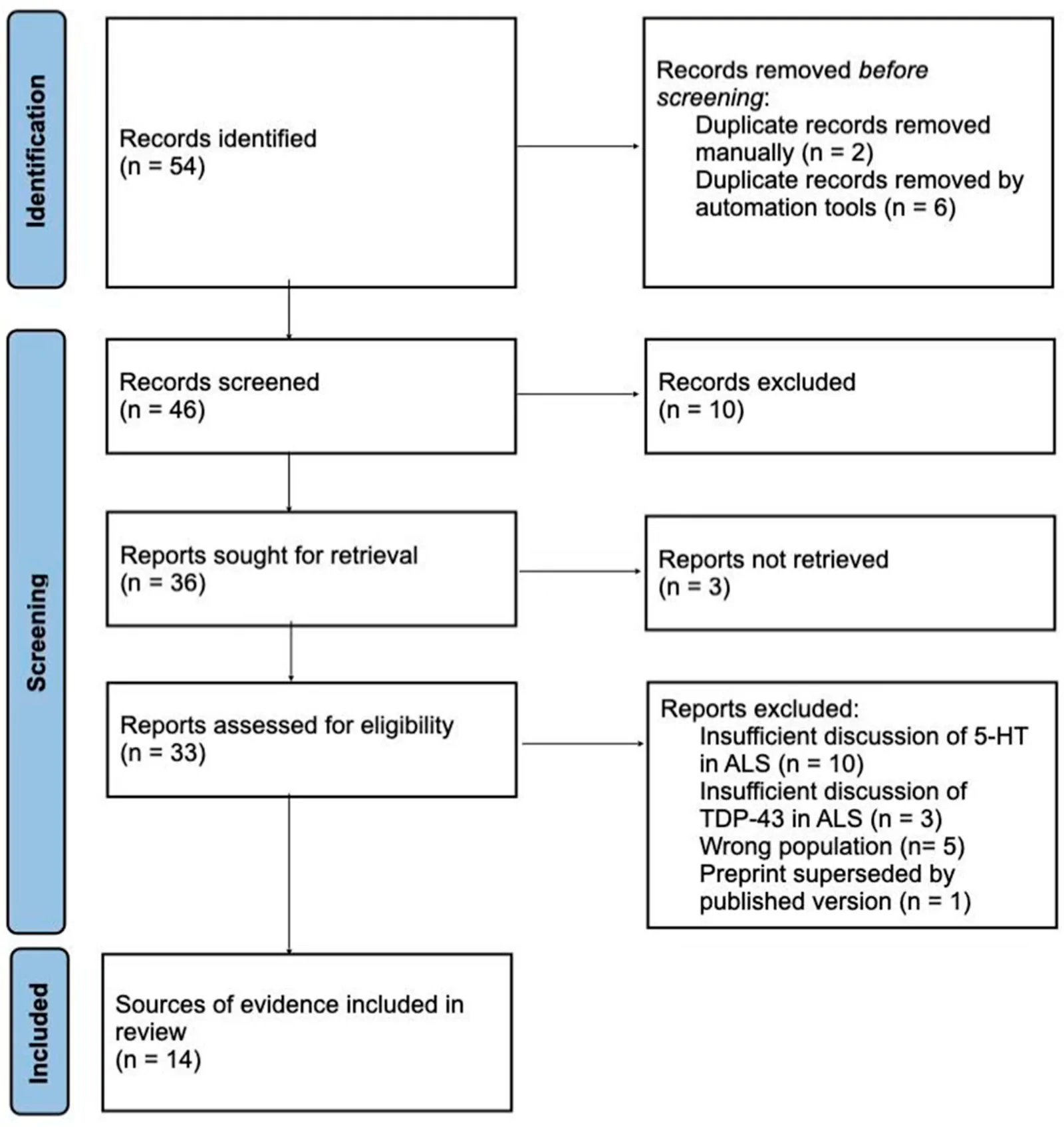
PRISMA 2020 flow diagram of study selection. Adapted from Page MJ et al. [19]. This work is licensed under CC BY 4.0. To view a copy of this license, visit https://creativecommons.org/licenses/by/4.0/ (accessed on 9 April 2026).

The electronic database search yielded a total of 46 records, as outlined in Figure 1. An additional 8 records were identified through snowball searching, for a total of 54 records. These records were imported into Covidence, where 6 duplicates were identified automatically and 2 manually, leaving 46 unique records for screening. Title and abstract screening resulted in the exclusion of 10 records. The remaining 36 records were sought for retrieval and eligibility assessment. Three full-text records could not be retrieved. An additional 19 records were excluded due to insufficient discussion of 5-HT in ALS (*n* = 10), insufficient discussion of TDP-43 in ALS (*n* = 3), wrong population (*n* = 5), and preprint superseded by published version (*n* = 1). The remaining 14 studies were included in this review.

### 3.2. Characteristics of Sources of Evidence

Across the 14 included sources of evidence, study designs included experimental animal studies, randomized and non-randomized analyses, and the review literature. Among the experimental studies, transgenic (Tg) mouse models were used most frequently, followed by *Caenorhabditis (C.) elegans* models, to elucidate specific cellular and mechanistic aspects of ALS in relation to serotonergic dysfunction and TDP-43 pathology. Review articles synthesized the existing literature on serotonergic signaling, including 5-HT receptor (5-HTR)-mediated mechanisms, (including those of specific 5-HTR subtypes, like 5-HT2B), cortical hyperexcitability, and potential therapeutic targets in ALS.

Included sources were published between 2012 and 2026, with more than half (57%) published between 2021 and 2026, reflecting growing research interest in this topic. Most papers originated from France (43%) and China (21%). Detailed characteristics of each source are presented in Table 1.

### 3.3. Results of Individual Sources of Evidence

The key findings from each study, presented in Table A1 (Appendix A), provide a comprehensive overview of the current evidence base.

### 3.4. Synthesis of Results

The synthesis of results was organized into three thematic groups: (1) the relationship between ALS and 5-HT, (2) the role of TDP-43 in ALS pathogenesis, and (3) the interaction between serotonergic mechanisms and TDP-43 in ALS. Findings across the 14 included studies were summarized within each theme to identify patterns, areas of convergence, and discrepancies in the literature. These papers identified distinct serotonergic alterations in ALS across multiple levels of neurobiological organization, including cellular, circuit, and systems levels.

## 4. Discussion

The findings of this review suggest that the relationship between serotonergic dysfunction and TDP-43 pathology may help elucidate the complex pathophysiology underlying ALS. Further experimental research is needed to clarify the links between serotonergic signaling and TDP-43 proteinopathy and to determine whether these pathways may represent viable therapeutic targets in ALS.

### 4.1. Summary of Evidence

#### 4.1.1. ALS and 5-HT

Available evidence supports an association between 5-HT and ALS, although the nature of this relationship remains incompletely defined. A consistent finding was the early and widespread impairment of central serotonergic systems, which appears to precede or accompany key pathological features of ALS rather than simply arise as a downstream consequence of motor neuron degeneration [20,21,22]. For the purposes of this review, serotonergic dysfunction refers to disruption of central 5-HT signaling at one or more levels of the serotonergic system, including alterations in 5-HT synthesis, availability, release or reuptake, degeneration of serotonergic neurons and their projections, and changes in the expression or function of specific 5-HTR subtypes [17,20,24]. In total, 12 of the 14 analyzed papers supported the presence of serotonergic impairment/dysfunction in ALS, while the remaining two described serotonergic system alterations without confirming a role in disease progression. Across these studies, no single biomarker nor diagnostic criterion for serotonergic dysfunction has been established; this is partially owing to the fact that 5-HT’s role in ALS is highly receptor-, dose-, and disease-stage-dependent rather than being adequately characterized by reduced 5-HT concentration alone [24,26,27,28]. In ALS, evidence supporting the broad idea of serotonergic dysfunction has been obtained using complementary and convergent biochemical, morphological, molecular, genetic and functional approaches. Within the included literature, biochemical techniques have identified alterations in 5-HT and its precursors and metabolites. These changes have been measured in cerebrospinal fluid, blood/platelet samples, and CNS tissue in humans, alongside high-performance liquid chromatography quantification of hypothalamic 5-HT levels in the mutant (m) *SOD1* mouse [17,24,25]. These findings demonstrate widespread functional disruption of serotonergic signaling in both ALS patients and disease models. Morphological analysis of human post-mortem ALS brain tissue has revealed extensive degeneration of central serotonergic neurons in the raphe superior central nucleus, while another region, the rostral medulla, shows relative preservation of neuronal cell bodies but with intense degeneration of their neurites [21]. In *SOD1*^G86R^ mice, molecular analysis further demonstrated downregulation of 5-HT transporter (Sert) and TPH2, the rate-limiting enzyme in neuronal 5-HT synthesis and a marker of serotonergic neurons, mRNA at symptom onset, while morphological TPH2 immunohistochemistry revealed atrophy of serotonergic neuronal cell bodies and neuritic degeneration [21]. Together, these findings provide convergent biochemical, morphological, and molecular evidence of serotonergic abnormalities in ALS, including evidence that some alterations occur before the onset of overt motor symptoms. Complementary functional/behavioural measures are also often utilised as a way to link altered serotonergic neuronal and receptor signalling to a distinct, measurable phenotype. One such study involving *SOD1*^G37R^ and conditional *SOD1*^G37R^ allele crossed with Tph2-Cre mice, used the long-lasting reflex (LLR), measured by tail electromyography, together with behavioural assessment of spasticity [23]. Additionally, they used a cell specific genetic manipulation to selectively delete m*SOD1* in brainstem 5-HT neurons [23]. By combining functional, genetic, and morphological approaches, researchers were able to characterize spasticity and motor phenotype in these mice. These studies further illustrate that serotonergic dysfunction is not readily captured by a single measure and instead requires multimodal lines of inquiry and evidentiary convergence. Moreover, there appears to be a role of genetic alteration in 5-HT signalling affecting ALS. It has been found that certain inherited polymorphisms in the human HTR2B (the gene that codes for the 5-HT2B) show increased levels of 5-HT2B mRNA in spinal cord, decreased degeneration of Iba1-positive cells (mononuclear phagocytes, including microglia), and slower disease progression [26]. Yang et al. [24] also discuss the broader context of modifier genes, noting that genetic variation can influence ALS phenotype without necessarily causing the disease itself.

One of the most consistent observations was a reduction in 5-HT levels and serotonergic innervation in both ALS patients and animal models [17,21,23,24]. Reduced platelet 5-HT levels in a subset of patients, coupled with a positive correlation with survival, suggests that serotonergic dysfunction may carry prognostic significance [17,20,24]. Because plasma 5-HT cannot cross the blood–brain barrier, decreased levels alone do not provide direct evidence of serotonergic dysfunction in the brain and spinal cord. However, it has been hypothesized that these peripheral findings may mirror serotonergic dysfunction within the CNS [22,25]. This interpretation has been supported by experimental data showing degeneration of serotonergic neurons in the RN, and a corresponding loss of their projections to the forebrain (hippocampus and hypothalamus) and to the spinal cord in ALS patients and ALS mouse models [21,22,23,25]. Importantly, in m*SOD1* mice, a loss of serotonergic function is found to occur presymptomatically, suggesting that this dysfunction may be an early contributor to ALS pathogenesis [20,21,24].

5-HT plays a key role in modulating motor neuron excitability, with alterations in the serotonergic system contributing to hyperexcitability and spasticity [20,22]. Several authors [20,21,22] generally agree that loss of serotonergic input leads to compensatory changes, including upregulation and constitutive activation of 5-HT2B/2C, which in turn increase motor neuron excitability [21,22]. Although this contributes to spasticity, El Oussini et al. [26] note it may also represent a compensatory mechanism, as reducing serotonergic-driven spasticity can worsen motor performance and accelerate disease progression in ALS models [20,23]. This highlights 5-HT’s multifaceted role, suggesting it may exert both protective and pathological effects.

5-HT may also be relevant to ALS through its relationship with glutamate-mediated excitotoxicity. Serotonergic denervation may reduce inhibitory control over glutamate release via presynaptic receptors, thereby exacerbating excitotoxic damage [17,24]. Brunet et al. [22] also highlights how 5-HT directly modulates persistent inward currents and calcium conductances in motor neurons, thereby contributing to sustained excitability. Together, these mechanisms position serotonergic dysfunction as a potential drive of excitatory-inhibitory imbalance observed in ALS.

##### Pharmacological Modulation of 5-HT Dysfunction in ALS

A substantial body of pharmacological research has attempted to clarify 5-HT’s role in ALS, but findings remain mixed. Jiang et al. [27] used three different 5-HTR antagonists to evaluate the effects of different receptor subtypes on disease progression. Interestingly, all three drug classes promoted the expression and/or the abnormal distribution of TDP-43 and *SOD1*^G93A^ proteins, but did not accelerate disease onset or shorten survival. These findings suggest a potential protective role for 5-HT in disease progression. Arnoux and Dupuis [20] noted that administration of 5-hydroxytryptophan (5-HTP) improves survival and locomotor function in m*SOD1* mouse models, demonstrating the therapeutic benefit of enhancing serotonergic tone and function. To build upon the promising effects of elevated central 5-HT, strategies aimed at reducing its clearance were evaluated, although with conflicting results. The selective 5-HT reuptake inhibitor (SSRI) fluoxetine, exacerbated disease progression in postnatal *SOD1*^G93A^ mice in a dose-dependent manner [22]. Notably, it has been posited that the deleterious effects of fluoxetine may be due to disrupted serotonergic neuron development during early exposure rather than 5-HT elevation itself [20]. Arnoux and Dupuis [20] also noted that monoamine oxidase-B (MAO-B) inhibition via high concentration or chronic rasagiline treatment has shown limited but suggestive clinical benefit, potentially through modulation of 5-HT metabolism. Altogether, these mixed pharmaceutical findings suggest that timing, dosage, and mechanism of serotonergic modulation may all be important. Further studies controlling for these factors may help identify future therapeutic targets, both presymptomatically and after symptom onset.

Only recently have different 5-HTR subtypes been investigated as exerting distinct effects in ALS, and this area warrants further study. Specifically, 5-HTR subtype 2B (5-HT2B) appears to mediate some of the protective effects of 5-HT in ALS as described by El Oussini et al. [26]. Global 5-HT depletion leads to the overexpression of 5-HT2B and subsequent constitutive receptor activity during the development of spasticity [21], suggesting a compensatory mechanism that may help preserve motor function [26]. El Oussini et al. [26] build on this by demonstrating that 5-HT2B upregulation in ALS Tg mouse models promotes microglial survival and slows disease progression, while its ablation significantly accelerates progression. In contrast, 5-HT2A signaling in astrocytes contributes to neuroinflammation and oxidative stress, particularly in later disease stages, where its inhibition improves motor neuron survival and extends lifespan in mouse models [28]. Jiang et al. [27] also evaluated the effects of 5-HTR subtypes 2, 3, and 4, finding that 5-HT3 inhibitors reduced body weight in Tg mice, whereas 5-HT2 and 5-HT4 inhibition worsened motor function. However, it is important to note that Jiang et al. [27] administered a pan-5-HT2 antagonist (ritanserin), which likely blocked the neuroprotective effects of upregulated 5-HT2B, and thereby abolished its ability to promote microglial survival. This loss of 5-HT2B signalling may have simultaneously negated the therapeutic benefit of 5-HT2A antagonism, which Lu et al. [28] demonstrated to be protective via suppressing oxidative stress, inhibiting astrocytic neuroinflammation and restoring autophagy with increased clearance of m*SOD1*. These differing results underscore the critical importance of investigating specific 5-HTR subtypes, because of their apparent opposing pathogenic versus protective roles. Table 2 details what is currently known about 5-HTR subtypes and how they may influence ALS pathogenesis.

Serotonergic dysfunction also extends beyond motor systems to influence metabolic and hypothalamic pathways. Reduced hypothalamic 5-HT levels and compensatory upregulation of 5-HT2C disrupts melanocortin signaling, contributing to altered feeding behaviour and hypermetabolism in ALS models [25]. Given that weight loss is associated with poorer prognosis, these findings highlight a broader systemic role for 5-HT in ALS progression.

Recent research supports a potentially protective role of 5-HT in disease progression, as the receptor antagonists studied in Jiang et al. [27] worsened several motor and disease-related features in Tg mouse models of ALS. This aligns with clinical observations that higher 5-HT levels are associated with reduced mortality risk [24]. However, the precise role of 5-HT remains complex, as both insufficient and excessive or mistimed, serotonergic signaling may be detrimental.

Table 3 details known modulators of 5-HT in the CNS along with their potential use in ALS treatments. It should be noted that the listed combination therapies remain untested and are based on preclinical evidence synthesized during this review process.

The pharmacological agents discussed above warrant further investigation, particularly to determine which specific 5-HTR subtype they target and whether their effects vary across stages of disease progression. It is of the authors’ opinion that elucidating the receptor-specific mechanisms of these agents, as well as the timing at which their effects are most pronounced, may provide insight into their potential to modulate ALS symptomatology and, ultimately, disease progression.

Taken together, these findings support a model in which ALS involves an early, progressive disruption of serotonergic systems, contributing to motor dysfunction, neuroinflammation, metabolic changes, and excitatory-inhibitory imbalances. 5-HT appears to exert both protective and pathological effects depending on receptor subtype, disease stage, and cell type, which likely explains the variability experimental and review-based results. Further work should prioritize targeted, receptor-specific investigation across different stages of disease in order to clarify the therapeutic potential of the serotonergic system in ALS.

#### 4.1.2. ALS and TDP-43

TDP-43 is a well-established pathological hallmark of several neurodegenerative diseases, including ALS and frontotemporal dementia (FTD). Compared with the emerging and more complex association between ALS and serotonergic dysfunction, the involvement of TDP-43 in ALS is relatively well established. Accordingly, our analysis focused primarily on whether the findings reported in our studies aligned with the broader body of literature. Overall, the extracted data were largely consistent with prior work.

TDP-43, encoded by the TARDBP gene, is a DNA/RNA-binding protein that plays a critical role in RNA metabolism, including transcription, splicing, and transport [33]. In ALS, pathological alterations in TDP-43 are observed in approximately 97% of cases, regardless of whether mutations in the TARDBP gene are present [20]. These alterations include mislocalization of TDP-43 from the nucleus to the cytoplasm, where it forms aggregates. This mislocalization leads both to loss of critical nuclear function and gain of toxic cytoplasmic function, contributing to widespread cellular dysfunction, including oxidative stress and impaired RNA processing [20,33]. Abnormal cytoplasmic TDP-43 is also believed to spread between cells through a prion-like process of seeding and propagation [7].

Beyond aggregation, several studies highlighted the broad metabolic and cellular consequences of TDP-43 dysfunction, much of which stems from loss of its essential nuclear functions. Disruption of RNA homeostasis, altered gene expression, and downstream effects on protein synthesis and cellular maintenance pathways collectively contribute to neuronal vulnerability and degeneration [20]. These mechanisms reinforce the central role of TDP-43 as a driver of cellular pathology in multiple regions of the nervous system in ALS, most prominently within motor-related areas.

Taken together, these findings reaffirm that TDP-43 proteinopathy is a unifying feature of ALS, characterized by largely consistent molecular, cellular, and anatomical patterns across studies. Although the core mechanisms of TDP-43 dysfunction are relatively well-established, its presence in regions associated with broader neuromodulatory systems highlights important avenues for further investigation, particularly in understanding how canonical ALS pathology may intersect with less-explored systems such as 5-HT.

#### 4.1.3. TDP-43 and 5-HT in ALS

Emerging evidence suggests that serotonergic dysfunction and TDP-43 proteinopathy are mechanistically interconnected in a complex and potentially bidirectional manner. Although this relationship remains incompletely defined, the studies reviewed here suggest a possible framework in which TDP-43 pathology may disrupt serotonergic signaling early in disease progression, while serotonergic dysfunction may in turn influence the propagation and severity of TDP-43 pathology.

Central to this interpretation is the possibility that TDP-43 pathology impairs serotonergic function at a cellular and synaptic level before overt neuronal loss occurs. In *C. elegans* models, expression of human TDP-43 variants produced graded impairments of 5-HT-dependent behaviours, with cytoplasmically mislocalized TDP-43 causing the most severe deficits [29]. These phenotypes suggest that pathological TDP-43 disrupts serotonergic signaling through functional impairment, such as altered neurotransmitter synthesis and release, or receptor signaling, rather than via complete degeneration of serotonergic neurons. This aligns with previous observations that TDP-43 may compromise neuromodulatory systems, including 5-HT, before neuronal death occurs [30]. Brunet et al. [22] further supports this interpretation by describing the effects of TDP-43 pathology on inhibitory synaptic transmission, which may also be linked to altered serotonergic function and destabilized motor circuits. At the systems level, these disruptions likely contribute to network dysfunction and imbalances between excitation and inhibition, a hallmark of ALS. Given 5-HT’s central role in modulating both excitatory and inhibitory inputs, it is reasonable to suspect that serotonergic dysfunction may amplify these effects. Indeed, Koopman et al. [31] demonstrated that motor circuit imbalance underlies TDP-43-induced paralysis, with altered neuromodulatory input contributing to reduced functional output. The authors noted potential serotonergic influence on cholinergic neurons, which may suppress their activity. Furthermore, this paper [31], highlighted how cellular changes caused by TDP-43 toxicity can render normal serotonergic signalling maladaptive. In their model, circuit dysfunction begins with TDP-43-induced degeneration of GABAergic neurons, disrupting motor circuit balance, while serotonergic signalling continues to suppress cholinergic motor neuron activity. In a balanced circuit, this serotonergic inhibition is necessary to regulate motor output; however, in a dysfunctional circuit, this suppression becomes detrimental and produces subsequent deficits in locomotion.

It is also important to consider that this interaction may operate in the reverse direction. Pharmacological evidence suggests that disruption of serotonergic signaling can exacerbate TDP-43 pathology. In ALS mouse models, administering 5-HTR antagonists increased TDP-43 expression and promoted its cytoplasmic mislocalization, increasing the number of TDP-43-positive cells [27]. Using Tg and wild-type (WT) mice, Jiang et al. [27] examined the effects of granisetron, piboserod, and ritanserin, antagonists for 5-HT3, 5-HT4, and 5-HT2, respectively. Antagonism of each receptor subtype consistently worsened TDP-43 pathology, increasing TDP-43 expression, accumulation, and cytoplasmic mislocalization, while also promoting neuroinflammation and neuronal loss; however, these treatments did not accelerate disease progression or shorten survival in mice. Altogether, these findings support the idea that 5-HT may play a protective or stabilizing role in maintaining TDP-43 homeostasis, and that loss of serotonergic tone may accelerate pathological aggregation processes [27].

This relationship is further complicated by cell-specific differences in vulnerability and pathology. In addition, while TDP-43 pathology is most commonly associated with motor systems, evidence indicates that its distribution extends beyond classical motor circuits. Dentel et al. [21] reported the presence of TDP-43 aggregates in regions with dense serotonergic innervation, such as the raphe magnus and gigantocellular nuclei. However, there is also evidence that serotonergic neurons themselves may be relatively resistant to typical TDP-43 aggregation. Vermeiren et al. [17] demonstrated that the upper RN which give rise to diffuse cortical projections, appear to be relatively spared from TDP-43 pathology in ALS, raising the possibility that specific serotonergic populations may exert protective effects or possess intrinsic resistance to protein aggregation. El Oussini et al. [23] similarly reported a lack of TDP-43 inclusions within serotonergic neurons, suggesting that these cells may possess unique mechanisms for clearing aggregation-prone proteins. Potential explanations for these discrepancies include regional differences within the RN, as well as differences among the models studied. Taken together, this evidence raises the possibility that serotonergic dysfunction in ALS arises more from functional impairment and circuit-level disruption than from intracellular aggregate toxicity within serotonergic neurons themselves.

The spatial propagation of TDP-43 pathology may also relate to this interpretation. Multiple studies support a prion-like spreading mechanism, in which misfolded and mislocalized TDP-43 propagates through neural networks over time [17,24]. Early environmental exposures may also trigger long-term neurodevelopmental vulnerabilities; for instance, neonatal exposure to the neurotoxin BMAA has been shown to produce persistent monoaminergic dysregulation and neuropathological changes characteristic of neurodegenerative disease [32]. Degeneration of serotonergic projections, or early deposition of pathological proteins within their target regions, may contribute to widespread monoaminergic imbalance, affecting not only motor function but also behavioural and cognitive domains [17]. There is reason to believe this prion-like propagation and serotonergic dysfunction are related, although the exact mechanism remains unresolved. As noted by Vercruysse et al. [25], 5-HT-related deficits, such as disruptions in melanocortin signaling, may influence, or be influenced by, the spread of TDP-43 aggregates, although the paucity of comparable research highlights an important gap in the literature.

In their discussion of melanocortin signaling, emerging work by Vercruysse et al. [25] points to a potential role of the gut–brain axis in linking these processes. Phosphorylated TDP-43 aggregates have been detected in the gastrointestinal tract before symptom onset, suggesting that ALS pathology may begin in the enteric nervous system and spread to the CNS [24]. Given that 5-HT is a key mediator of the gut–brain–microbiome axis, this raises the intriguing possibility that peripheral serotonergic signaling may influence early TDP-43 pathology or its propagation. If confirmed, such a mechanism would further support a systemic, rather than purely central, view of ALS pathogenesis.

**Figure 2:**
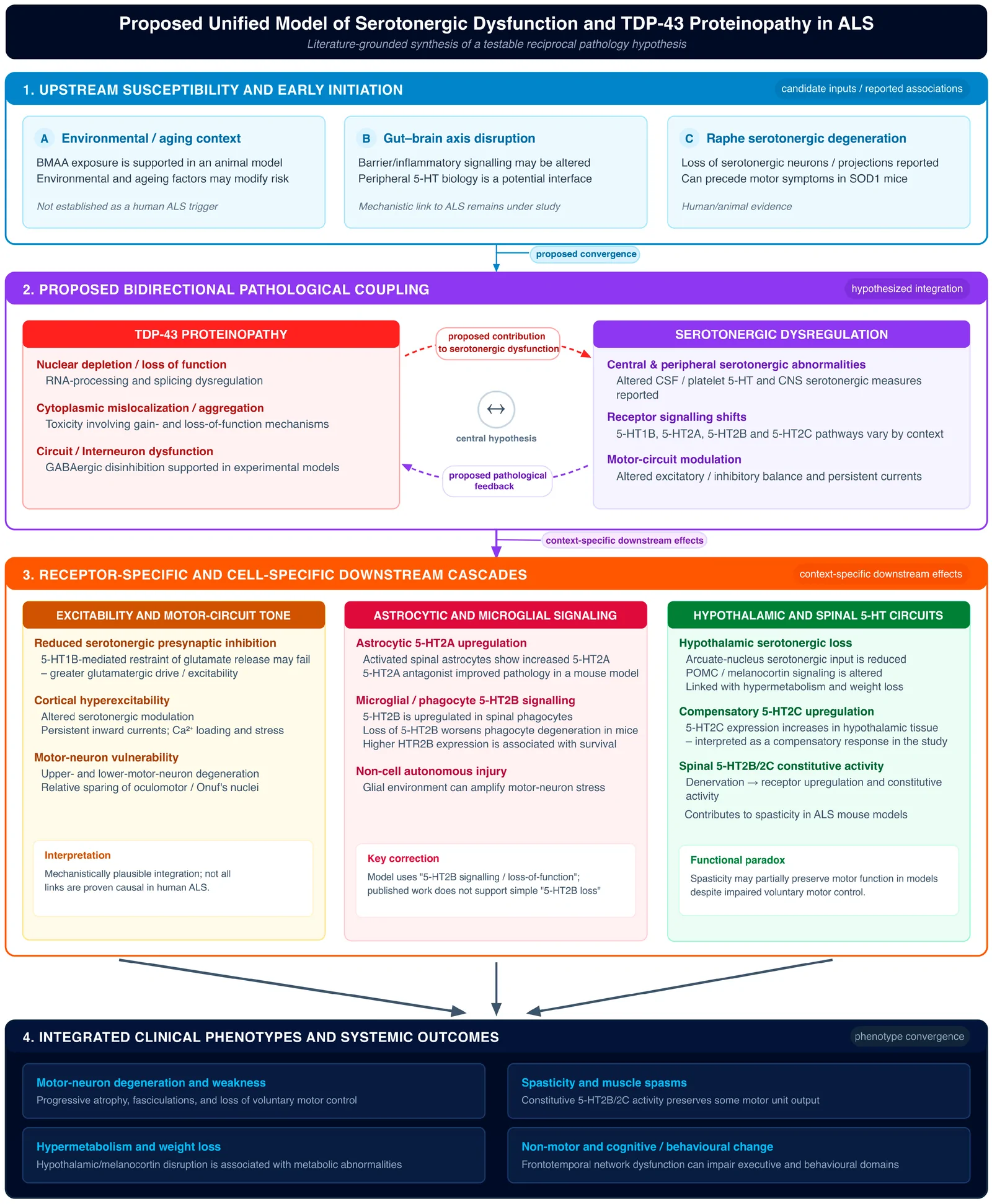
Proposed unified pathogenetic pathway of 5-HT dysfunction and TDP-43 proteinopathy in ALS. Data adapted from [1,6,7,8,9,10,14,17,20,21,22,23,24,25,26,28,30,32,33].

Taken together, these multifaceted findings support a model in which TDP-43 proteinopathy and serotonergic dysfunction are interconnected across multiple levels of disease progression. TDP-43 may disrupt serotonergic signaling early through synaptic and molecular mechanisms, thereby contributing to circuit dysfunction and hyperexcitability. Conversely, serotonergic deficits may exacerbate TDP-43 aggregation and propagation, either by removing protective influences or by altering cellular homeostasis. The apparent resistance of serotonergic neurons to protein aggregation, despite functional impairment, further underscores the complexity of this relationship. A comprehensive diagram summarizing the proposed bidirectional and interactive relationship among ALS, 5-HT, and TDP-43 is provided in Figure 2.

Overall, this potential bidirectional relationship highlights the need to move beyond single-pathway explanations of ALS and adopt a more integrated framework in which protein aggregation, neuromodulatory dysfunction, and network-level disturbances converge to drive disease progression. Future research should prioritize clarification of the mechanistic links between TDP-43 and 5-HT, particularly in relation to protein propagation, synaptic regulation, and the gut–brain axis, in order to better inform the development of targeted and temporally precise therapeutic strategies.

#### 4.1.4. Translational Medicine

This emerging relationship between serotonergic dysfunction and TDP-43 pathology has potential implications for translational research in ALS. Although the evidence remains predominantly preclinical and indirect, the findings reviewed suggest that serotonergic signaling may represent a clinically relevant pathway through which molecular pathology (i.e., TDP-43 pathologies), neuronal excitability, neuroinflammation, and central serotonergic dysfunction converge. Importantly, the complex and context specific constitution of 5-HT suggest that therapeutic strategies aimed at broadly increasing or decreasing serotonergic signalling may be insufficient and, in some instances, potentially detrimental. This may partly explain the limited and occasionally adverse effects observed with SSRI treatment: chronic fluoxetine administration did not alter disease progression in adult *SOD1*^G93A^ mice, whereas high-dose neonatal treatment accelerated motor decline and shortened lifespan [22,24]. Future translational approaches may instead benefit from targeting specific serotonergic receptor subtypes, cell populations, or downstream signaling pathways, while also accounting for the stage of the disease.

Based on the preclinical evidence presented here, it is the authors’ opinion that serotonergic interventions may be most efficacious during presymptomatic and/or early symptomatic stages of ALS when serotonergic abnormalities may already be present, but extensive neurodegeneration is yet to occur. However, current evidence is insufficient to establish whether intervention at these stages would be impactful in human populations. The authors propose additional translational research into serotonergic treatments with careful consideration into the unique 5-HTR subtype mechanisms. It should also be noted that potential benefit of intervention in early-stage sALS has not been demonstrated, and treating sALS in the presymptomatic stage is not feasible. As such, we propose that at risk populations, such as individuals carrying known ALS-associated genetic mutations, be included in longitudinal studies aimed at determining the stage at which serotonergic abnormalities arise relative to symptom onset and whether they represent a viable target for early intervention.

Further, a potential application is the development of biomarkers that identify serotonergic dysfunction and its relationship to TDP-43 pathology. At present serotonergic abnormalities may only be detected through multi-model inquiry and/or through indirect measurement of peripheral markers (such as platelet 5-HT levels) [17,20,21,22,23,24,25]. However, no validated biomarker currently captures serotonergic dysfunction in ALS, or establishes its relationship to disease progression. Combining measures of serotonergic function with established or emerging biomarkers of TDP-43 pathology could help determine the linearity of serotonergic abnormalities and whether their precede, accompany, or follow TDP-43 pathologies. Such approaches may also facilitate patient stratification in clinical trials by identifying individuals with greater serotonergic involvement who may be more likely to respond to serotonergic-focused interventions.

The potential interaction between 5-HT and TDP-43 also probes for investigation of combination or pathway-directed therapies. If serotonergic dysfunction is contributing to TDP-43 mislocalization or impaired clearance, restoring specific serotonergic signaling pathways could theoretically support approaches aimed at reducing TDP-43 pathology. Conversely, if TDP-43 pathology is disrupting serotonergic signaling as an early disease consequence, serotonergic modulation may provide a means of preserving circuit function, despite ongoing cellular pathology. Currently, these possibilities remain hypothetical, and require validation via experimental models that simultaneously measure serotonergic signaling, TDP-43 pathology, neuronal function, disease progression, and clinical phenotypes. However, examining these pathways together may provide a more holistic framework for therapeutic development than investigating either pathway independently.

The evidence reviewed in this paper supports several priorities for future translational ALS research: establishing clinically relevant biomarkers of serotonergic dysfunction, defining receptor- and cell-specific mechanisms, determining how these pathways change throughout disease progression, and testing whether modulation of 5-HT signaling alters TDP-43 pathology. It is important to highlight that the current evidence does not establish 5-HT modulation as an effective treatment for ALS. Instead, it identifies a biologically plausible and relatively underexplored pathway that may connect TDP-43 proteinopathy with neuronal excitability, neuroinflammation, metabolism, and circuit dysfunction. Clarifying this relationship could contribute to valuable biomarker development and therapuetically targeted approaches to ALS.

### 4.2. Limitations

As a scoping review, this study has several limitations. It was not designed to provide definitive or causal conclusions, but rather to synthesize the existing evidence and identify gaps in a complex and underexplored area. Although data extraction was conducted by two independent reviewers, the small number of reviewers introduces a risk of selection and interpretation bias. In addition, the limited inclusion of the grey literature may contribute to publication bias, as unpublished or negative findings are likely underrepresented. There is also potential for search and selection bias related to database limitations, keyword choices, and possible exclusion of relevant studies. Moreover, this review did not include a formal critical appraisal of included sources of evidence (PRISMA-ScR Item 12). Although omission of critical appraisal is consistent with PRISMA-ScR guidance for scoping reviews, the lack of quality assessment limits the ability to evaluate the methodological rigor and risk of bias of the included studies. Considerable heterogeneity across studies, including differences in experimental models (human, mouse, and *C. elegans*), methodologies, and outcome measures, limits direct comparison and may reduce generalizability. This is particularly relevant because many mechanistic insights are derived from animal models, which may not fully reflect human ALS pathology. Finally, the relative scarcity of studies directly examining interactions between 5-HT and TDP-43 necessitates reliance on indirect evidence, which may introduce interpretive assumptions when proposing mechanistic links.

## 5. Conclusions

Although complex and multifaceted, this review highlights a potential relationship between TDP-43 proteinopathy and serotonergic dysfunction in ALS. Further experimental research is needed to clarify the nature and directionality of this relationship, particularly with careful consideration of disease stage and model system (e.g., *C. elegans*, rodent, or human patient). A more precise understanding of the relationship between 5-HT and TDP-43 may help identify novel therapeutic targets, potentially including the gut–brain axis or more specific 5-HTR subtypes (e.g., 5-HT1A, 5-HT2B).

## Figures and Tables

**Table 1:** Characteristics of included sources of evidence.

Author & Year	Ref.	Location	Study Design	Model/Population	Main Focus
Vermeiren et al. (2018)	[17]	Belgium	Review	N/A	Serotonergic dysfunction in ALS and Parkinson’s disease
Arnoux & Dupuis (2021)	[20]	France	Book chapter	N/A	Synthesize current knowledge of 5-HT2B in ALS
Dentel et al. (2013)	[21]	France	Non-randomized experimental animal study	Mouse ALS model	Linking serotonergic neuron degeneration in ALS to spasticity
Brunet et al. (2020)	[22]	France	Review	N/A	Cellular and molecular origins of cortical hyperexcitability in ALS
El Oussini et al. (2017)	[23]	France	Non-randomized experimental animal study	Mouse ALS model	Providing evidence for brainstem 5-HT neurons’ role in spasticity
Yang et al. (2023)	[24]	China	Review	N/A	Summarizing current knowledge on serotonergic alterations in ALS
Vercruysse et al. (2016)	[25]	France	Secondary analysis of RCT and non-randomized experimental animal study	N/A	Hypothalamic melanocortin pathway alterations in ALS
El Oussini et al. (2016)	[26]	France	Non-randomized experimental animal study	Mouse ALS model	5-HT2B’s effects on ALS disease progression
Jiang et al. (2023)	[27]	China	Non-randomized experimental animal study	Mouse ALS model	The relationship between ALS and 5-HT, using 5-HTR antagonists
Lu et al. (2024)	[28]	China	Non-randomized experimental animal study	Mouse ALS model	Therapeutic effects of desloratadine (DLT), a 5-HT2A antagonist
Lacour et al. (2026)	[29]	Argentina	Non-randomized experimental study	*C. elegans* model	Potential graded severity of TDP-43 effects in 5-HT neuron-specific *C. elegans*
Loh et al. (2024)	[30]	Malaysia	Review	N/A	The role of the microbiota–gut–brain axis in neurodegenerative diseases
Koopman et al. (2024)	[31]	Netherlands	Non-randomized experimental study	*C. elegans* model	Biological mechanisms for disease-related phenotypes in ALS
Scott & Downing (2018)	[32]	South Africa	Non-randomized experimental animal study	Mouse ALS model	BMAA exposure and neurodegeneration relevant to ALS

Regarding the financial sponsorship of the included literature, public funding accounted for 92.9% (13/14) of the analyzed studies, while one study (7.1%) did not report a funding source. No industry-sponsored or non-sponsored papers were identified.

**Table 2:** 5-HTR Family Signaling Pathways and Pathological Roles in ALS.

Receptor Subtype	Type	Location	Mechanism of Action	Downstream Cascade and Role in Pathogenesis/Protection
5-HT2B	Gq11-protein coupled	Microglia and mononuclear phagocytes [26]	Increasing intracellular concentration of IP3 and DAG	Protective: Upregulation of 5-HT2B to down-stream MAPK/ERK1/2 signaling pathways mediate microglial survival and anti-inflammatory function [26].
5-HT_3_	Ligand-gated Na+/K+ channel	GABAergic interneurons and glutamatergic neurons	Depolarization of cell plasma membrane	Homeostatic: 5-HT3 blockade with granisetron increased total spinal TDP-43 and *SOD1*^G93A^, increased TDP-43-positive cells and cytoplasmic TDP-43 localization, increased astrocytes in selected spinal regions, and reduced neuronal numbers in anterior horn/central-canal regions. It also worsened body-weight loss, but did not significantly alter hanging-wire strength or ALS progression [27].
5-HT_4_	Gs-protein coupled	CNS neurons	Increasing intracellular concentration of cAMP	Protective: 5-HT4 blockade increased spinal TDP-43 and m*SOD1* levels, TDP-43 cytoplasmic localization, microglial abundance in selected regions, and neuronal loss. Hanging-wire performance deteriorated, although global disease progression was unchanged [27]. Suggests a protective 5-HT4-dependent signal, potentially particularly relevant to microglia and mutant-*SOD1* handling, but again this rests largely on one *SOD1*^G93A^ study. No convincing ALS-model 5-HT4 agonist rescue experiment has yet established causality.
5-HT2A	Gq11-protein coupled	Spinal astrocytes [28]	Increasing intracellular concentration of IP3 and DAG	Pathogenic: Upregulation and activation of 5-HT2A leads to astrocytic activation triggering neuroinflammation and reduced autophagic clearance as well as neuronal calcium influx culminating in excitotoxic cell death [28].

IP3: inositol trisphosphate; DAG: diacylglycerol; cAMP: cyclic adenosine monophosphate. Note: Canonical receptor coupling and channel biophysics reflect standard GPCR pharmacology [14,16]; cell-type expression profiles and disease-specific mechanisms in ALS are cited from the primary literature [26,27,28].

**Table 3:** Pharmacological Modulators of Central Serotonergic Signaling and Their Effects in ALS.

Pharmacological Agent	Serotonergic Target	Evidence in ALS	Potential Therapeutic Relevance	Reference
Dextromethorphan/quinidine (DM/Q)	Inhibits SERT and reduced 5-HT reuptake and can increase extracellular 5-HT	Treatment of pseudobulbar affect (PBA) and impaired emotional control, with dextromethorphan modulating serotonergic signaling and quinidine increasing its bioavailability through inhibition of CYP2D6.	Has already demonstrated clinical efficacy in reducing PBA in human ALS population. Benefits of this treatment are likely due to combinatorial serotonergic, sigma-1 and NMDA mechanism.	[12]
5-Hydroxytryptophan (5-HTP)	Metabolic precursor of 5-HT; increases endogenous 5-HT synthesis	In presymptomatic *SOD1*^G93A^ mice, 5-HTP delayed hindlimb weakness and mortality and improved locomotor function and survival.	Directly addresses reduced serotonergic availability; beneficial motor and survival effects reported preclinically.	[20,24]
Fluoxetine	Binds to the 5-HT transporter (SERT) and blocks the reuptake of 5-HT; indirectly increases extracellular 5-HT	Transient neonatal administration of fluoxetine let to a decrease in weight, and an increased rate of motor impairment and exhibited accelerated mortality in *SOD1*^G93A^ mice. Separately, acute fluoxetine treatment in *SOD1*^G86R^ mice restored hypothalamic *Pomc* expression and reversed hyperphagia associated with serotonergic loss.	Suggests that increasing serotonergic tone can normalize a specific hypothalamic/metabolic phenotype, while the adverse effects of neonatal exposure highlight the importance of intact serotonergic development and treatment timing.	[22,24,25]
SB206553	5-HT2B/2C receptor inverse agonist	In m*SOD1* murine models with loss of serotonergic input and compensatory 5-HT2B/2C activity, SB206553 reduced/abolished spasticity.	May be able to treat painful ALS symptomatology such as hyperreflexia and spasticity; however, caution is warranted because spasticity may partially compensate for declining motor function	[21,23,24]
Granisetron	Selectively inhibits 5-HT3	Reduced body weight and increased TDP-43/*SOD1*^G93A^ abnormalities and glial changes in *SOD1*^G93A^ mice; it did not improve disease progression	Suggests endogenous 5-HT3 signaling may restrain aspects of TDP-43 dysregulation/astrogliosis, but evidence presently limited to one ALS study and has not established the downstream mechanism.	[27]
Piboserod	High-potency 5-HT4 antagonist	Worsened motor performance and increased pathological TDP-43/*SOD1*^G93A^ measures in *SOD1*^G93A^ mice.	Useful in identifying 5-HT4 signaling as potentially protective.	[27]
Ritanserin	High-potency 5-HT2 antagonist	Worsened motor performance and increased pathological protein abnormalities and cellular pathology in *SOD1*^G93A^ mice.	Demonstrates that indiscriminate 5-HT2 blockade may be detrimental and that receptor subtype/cell type should be considered when designing serotonergic therapies.	[27]
Desloratadine (DLT)	5-HT2A antagonist (in addition to its established H_1_-antihistamine activity)	Treatment of *SOD1*^G93A^ mice delayed symptom onset, prolonged lifespan, improved motor performance and reduced motor-neuron pathology. Effects were linked to spinal astrocytic 5-HT2A signaling.	Further supports 5-HT2A signalling as being pathogenic. The study also links a defined serotonergic target to astrocyte activation, autophagy, oxidative stress and neuroinflammation.	[28]

## Data Availability

No new data were created or analyzed in this study. Data sharing is not applicable to this article. All data and search strategies supporting the findings of this review are included within the manuscript and its Appendix A.

## Supplementary Materials

The following supporting information can be downloaded at: https://www.mdpi.com/article/10.3390/biom16101470/s1. Supplementary File S1: Preferred Reporting Items for Systematic reviews and Meta-Analyses extension for Scoping Reviews (PRISMA-ScR) [18]. Supplementary File S2: Search strategy.

## Appendix A

**Table A1:** Results of individual sources of evidence.

Author & Year	Ref.	Key Findings
Vermeiren et al. (2018)	[17]	Reviewed evidence of serotonergic dysfunction in ALS and found reduced 5-HT levels, altered 5-HTR expression, degeneration of serotonergic pathways, and associations between platelet 5-HT levels and patient survival. They proposed that loss of serotonergic inhibition, particularly through 5-HT1B, may increase glutamate release and exacerbate excitotoxic motor neuron damage. The review also highlighted neuroprotective roles of 5-HT through glial 5-HT1A and 5-HT2B signaling and discussed the relative sparing of upper RN from TDP-43 pathology. Overall, serotonergic dysfunction was proposed to contribute to ALS progression through excitotoxicity, metabolic dysregulation, and impaired neuroprotection, although no direct mechanism linking 5-HT to TDP-43 aggregation was identified.
Arnoux & Dupuis (2021)	[20]	Presented evidence for the dual role of serotonergic signaling in ALS disease progression, emphasizing that not all 5-HTR subtypes are uniformly beneficial or detrimental. They noted that 5-HT2A expressed on pyramidal cells (including motor neurons) may be responsible for motor neurons’ sensitivity to glutamate-mediated excitation and potentially contribute to excitotoxic mechanisms implicated in ALS. In contrast, they presented evidence supporting a protective role of the 5-HT2B and its predominant expression on microglia and mononuclear phagocytes, where it may modulate neuroinflammation and slow disease progression.
Dentel et al. (2013)	[21]	Authors countered the traditional view that ALS associated spasticity arises solely as a result of upper motor neuron (corticofugal pathway) degeneration and propose that 5-HT-producing cells and their projections to the spinal cord and hippocampus deteriorate significantly in both human patients and animal models. Using a mouse model, the researchers discovered that 5-HT levels drop prior to the appearance of motor symptoms, leading to a compensatory increase in 5-HT2B expression thus leading to spasticity. Additionally, they introduce experimental evidence showing that inverse agonists for 5-HT2B/2C effectively eliminated spasticity in mouse models. Moreover, they suggest that medications like cyproheptadine should undergo rigorous trials but caution that treating spasticity may worsen motor function, as seen in patients with spinal cord injury.
Brunet et al. (2020)	[22]	This review focused on the cortical hyperexcitability present in early-stage ALS, proposing that it may support a cortical origin of ALS and related neurodegenerative diseases. They presented evidence that decrease in cortical 5-HT, as seen in ALS models, may be accompanied by the upregulation of constitutively active serotonergic receptors, which may contribute to increased cortical excitability. Additionally, the authors presented evidence linking cortical hyperexcitability with increased TDP-43 immunoreactivity, thus offering an indirect association between 5-HT mediated neuronal excitation and TDP-43 pathology.
El Oussini et al. (2017)	[23]	Demonstrated that descending bulbospinal neuron degeneration is necessary for the development of spasticity in ALS. They used a conditional m*SOD1* mouse model and showed that selectively preventing m*SOD1* expression in 5-HT neurons preserved spinal serotonergic innervation, prevented constitutive 5-HT2B/2C activity, and abolished spasticity. However, ‘rescuing’ serotonergic neurons worsened motor function and accelerated paralysis onset, suggesting that spasticity may partially compensate for motor deficits. Additionally, consistent with previous observations of a lack of TDP-43 aggregates in serotonergic neurons from ALS patients, m*SOD1* mouse models also exhibited very few *SOD1*-positive aggregate-containing neurons, suggesting that serotonergic neurons may have distinct clearance mechanisms that protect against protein aggregation.
Yang et al. (2023)	[24]	Highlighted evidence indicating that serotonergic dysfunction is a common feature of ALS. Reduced 5-HT and tryptophan levels, altered 5-HTR expression, and degeneration of serotonergic pathways were associated with disease severity and progression. Proposed mechanisms include increased glutamate-mediated excitotoxicity, altered motor neuron excitability, and dysregulated neuroinflammation. The review also discussed emerging links between the gut–brain axis, TDP-43 pathology, and serotonergic signaling, suggesting potential interactions that warrant further investigation.
Vercruysse et al. (2016)	[25]	Investigated alterations in the hypothalamic melanocortin pathway in ALS using m*SOD1* and TDP-43 mouse models, as well as ALS patient samples. They found reduced hypothalamic 5-HT levels, loss of serotonergic innervation in the arcuate nucleus, and increased 5-HT2C expression, suggesting compensatory responses to diminished serotonergic input. Because 5-HT normally stimulates proopiomelanocortin (Pomc) signaling through 5-HT2C, this serotonergic deficit disrupted melanocortin-mediated regulation of feeding behaviour. Treatment with fluoxetine restored Pomc expression and normalized feeding abnormalities, supporting a functional role for 5-HT in these metabolic disturbances. Similar alterations were observed in both m*SOD1* and TDP-43 models, suggesting that hypothalamic serotonergic dysfunction may represent a common feature of ALS pathology independent of the underlying genetic cause.
El Oussini et al. (2016)	[26]	Identified 5-HT2B to be implicated not only in the development of spasticity, but also as a disease modifier. They presented evidence that functional 5-HT2B may be protective against mononuclear phagocyte degeneration. They provided further experimental evidence by ablating 5-HT2B in *SOD1*^G86R^ mice, which reduced their survival by 30%. They suggested that the dramatically reduced survival time was because of its ablation on mononuclear phagocytes. In a human ALS cohort, the researchers identified a genetic variant associated with higher *HTR2B* expression and thereby more 5-HT2B synthesis. This was associated with greater preservation of Iba1 positive cells (microglia and macrophages) and increased survival time, supporting a protective role for 5-HT2B signaling in ALS progression.
Jiang et al. (2023)	[27]	Using *SOD1*^G93A^ Tg ALS and WT mice, researchers administered three different 5-HTR antagonists: granisetron, piboserod, and ritanserin, which target 5-HT3, 5-HT4, and 5-HT2, respectively. They found that each antagonist exacerbated ALS-related pathology and symptoms. Granisetron significantly reduced body weight in Tg mice, while piboserod and ritanserin worsened motor function. Importantly, all three antagonists increased the expression of TDP-43 and *SOD1* proteins and accelerated the mislocalization of TDP-43 from the nucleus to the cytoplasm. The authors suggested that 5-HT deficiency may contribute to the pathogenesis of ALS by altering the expression and distribution of TDP-43 and *SOD1*, as well as by promoting glial activation.
Lu et al. (2024)	[28]	Demonstrated that in *SOD1*^G93A^ ALS mice, spinal astrocytes showed increased 5-HT2A expression. Pharmacological (desloratadine) or genetic inhibition of 5-HT2A delayed symptom onset, prolonged survival, reduced motor neuron loss, enhanced autophagy, and decreased oxidative stress and neuroinflammation. This suggests that excessive 5-HT2A signaling contributes to ALS pathology and that 5-HT2A antagonism is neuroprotective.
Lacour et al. (2026)	[29]	Developed a serotonergic neuron-specific *C. elegans* model expressing cytoplasmic human TDP-43 to investigate the effects of TDP-43 pathology on serotonergic function. They found that mislocalized TDP-43 caused early behavioural and locomotor impairments despite the absence of overt neurodegeneration or neuronal loss. These findings suggest that TDP-43 pathology can disrupt serotonergic neuron function before structural degeneration occurs. This supports the idea that early serotonergic dysfunction may contribute to disease-related symptoms independently of neurodegeneration.
Loh et al. (2024)	[30]	Noted that roughly 90% of 5-HT is produced by gut enterochromaffin cells, that gut microbiota can affect hippocampal 5-HT, and that increased intestinal 5-HT can reshape gut bacterial colonization in a bidirectional host–microbial loop. Changes in gut bacteria are said to potentially weaken the intestinal barrier and blood–brain barrier, increase inflammation, and worsen diseases, including ALS. TDP-43 was discussed in this paper as a key biomarker of disease progression, consistent with previous research, and ALS iPSCs carrying the TDP-43 G298S mutation showed rescued mitochondrial respiratory defects following nicotinamide treatment.
Koopman et al. (2024)	[31]	Utilized a *C. elegans* model expressing human TDP-43 and found that TDP-43 caused degeneration of GABAergic neurons and impaired cholinergic motor neuron function, resulting in an imbalanced motor circuit. Using a phenomics profile, they identified 5-HTR signaling as a major pathway contributing to the behavioural and locomotor defects observed in TDP-43-expressing worms. Pharmacological inhibition of 5-HTRs with mianserin or methiothepin improved acetylcholine-dependent neurotransmission and restored movement, whereas exogenous 5-HT exacerbated paralysis. 5-HTR signaling is highlighted as a maladaptive contributor to motor circuit dysfunction, whereby its normally inhibitory role becomes detrimental when the circuit is compromised by TDP-43 toxicity.
Scott & Downing (2018)	[32]	Examined the long-term effects of a single neonatal exposure to β-N-methylamino-L-alanine (BMAA) in a rat model of ALS/parkinsonism-dementia complex. They found persistent alterations in serotonergic and dopaminergic signaling, together with neuropathological changes characteristic of neurodegenerative disease, including spinal cord TDP-43 pathology. The authors proposed that disruption of 5-HT and dopamine signaling during critical developmental periods may increase vulnerability to later neurodegeneration by altering neuronal development and circuit formation. The coexistence of serotonergic abnormalities and TDP-43 pathology suggests that early neurotransmitter dysregulation may contribute to pathways associated with ALS-related neurodegeneration. The study suggests that early serotonergic dysfunction may represent an upstream factor contributing to neurodegenerative mechanisms associated with TDP-43 pathology.
