# Supplementary material for: Serotonergic Dysfunction and the Interacting Role of TAR DNA-Binding Protein 43 in the Progression of Amyotrophic Lateral Sclerosis: A Scoping Review
Source: Biomolecules. 2026 Oct 9;16(10):1470. doi: 10.3390/biom16101470 (PMC13702111; doi:10.3390/biom16101470)
Supplement: Supplementary file 1 [file biomolecules-16-01470-s001.zip › Supplementary File S2.pdf]

Supplementary File S2 – Search Strategy

<u>OVID – Medline</u>

Search terms:

(amyotrophic lateral sclerosis or sclerosis, amyotrophic lateral or als -alsgehrig's disease or gehrig disease or gehrigs disease or gehrig's disease or lou gehrig disease or motor neuron disease or lou gehrig's disease or motor neurone disease or lou-gehrigs disease or disease, lou-gehrigs or charcot disease or amyotrophic lateral sclerosis with dementia dementia with amyotrophic lateral or sclerosis, guam form or guam disease or disease, guam or amyotrophic lateral sclerosis-parkinsonism-dementia complex 1 or amyotrophic lateral sclerosis parkinsonism dementia complex 1 or amyotrophic lateral sclerosis, parkinsonism-dementia complex of guam or amyotrophic lateral sclerosis, parkinsonism dementia complex of guam).mp.

(serotonin or 5-ht or 5-hydroxytryptamine or 5 hydroxytryptamine or 3-2-aminoethyl-1h-indol-5-ol or enteramine or hydroxytryptamine or 50-67-9 or 1H-Indol-5-01, 3-2-aminoethyl- or receptors, serotonin or serotonin receptor or receptor, serotonin or 5-ht receptor or 5 ht receptor or receptor, 5-ht or receptors, 5-hydroxytryptamine or receptors, 5 hydroxytryptamine or 5-hydroxytryptamine receptors or 5 hydroxytryptamine receptors or receptors, 5-ht or receptors, 5 ht or 5-ht receptors or 5 ht receptors or 5-hydroxytryptamine receptor or 5 hydroxytryptamine receptor or receptor, 5-hydroxytryptamine or receptors, tryptamine or tryptamine receptors or tryptamine receptor or receptor, tryptamine).mp.

(tdp-43 proteinopathies or proteinopathies, tdp-43 or proteinopathy, tdp-43 or tdp 43 proteinopathies or tdp-43 proteinopathy or TDP-43 or TDP43 or TARDBP or TAR DNA-binding protein 43 or TAR DNA-binding protein-43 or ALS10 or TARDBP or TAR DNA binding protein or ALS10 or Transactive response DNA binding protein 43 kDa or protein TDP 43 or protein TDP43 or TAR DNA binding protein 43 or TDP 43 protein or TDP43 protein or TAR DNA binding protein).mp.

1 and 2 and 3

Limits:

None
